# Ross River Virus Disease Reemergence, Fiji, 2003–2004

**DOI:** 10.3201/eid1104.041070

**Published:** 2005-04

**Authors:** Philipp Klapsing, J. Dick MacLean, Sarah Glaze, Karen L. McClean, Michael A. Drebot, Robert S. Lanciotti, Grant L. Campbell

**Affiliations:** * Montreal General Hospital, Montreal, Quebec, Canada;; † McGill University Health Centre, Montreal, Quebec, Canada;; ‡ Royal University Hospital, Saskatoon, Saskatchewan, Canada;; § Health Canada, Winnipeg, Manitoba, Canada;; ¶ Centers for Disease Control and Prevention, Fort Collins, Colorado, USA

**Keywords:** Ross River virus, arbovirus, alphavirus, epidemic polyarthritis, travel, Fiji, dispatch

## Abstract

We report 2 clinically characteristic and serologically positive cases of Ross River virus infection in Canadian tourists who visited Fiji in late 2003 and early 2004. This report suggests that Ross River virus is once again circulating in Fiji, where it apparently disappeared after causing an epidemic in 1979 to 1980.

The growing appreciation of travelers as sentinels for the emergence of infectious diseases is based on the immunologic naiveté of travelers, their defined exposure in time and space, and sufficient diagnostic resources after their return to an industrialized country. Reports of infectious diseases in travelers in unusual numbers or from new geographic locations can inform a public health response. We report 2 recent apparent cases of Ross River virus disease ("epidemic polyarthritis") in Canadian travelers to Fiji, ≈1,000 miles from the region (Australia, New Guinea, and the Solomon Islands) where the virus is endemic, enzootic, and often epidemic (*1*).

Ross River virus, a mosquitoborne alphavirus in the family *Togaviridae*, is a single-stranded, enveloped RNA virus. Other viruses in this family include Chikungunya, o'nyong-nyong, Sindbis, and eastern and western equine encephalitis. In Australia, the major vectors of Ross River virus to humans are various *Culex* and *Aedes* mosquitoes. Marsupials (especially kangaroos and wallabies) are the most important vertebrate amplifying hosts (*1*). Several thousand cases of epidemic polyarthritis are reported annually in Australia, making Ross River virus the most important arboviral pathogen in that country (*2**,**3*). In 1979, Ross River virus spread dramatically to the South Pacific islands (probably imported by a viremic person arriving from Australia), including Fiji, American Samoa, Wallis and the Cook Islands, causing the largest Ross River virus epidemic ever recorded (*4**–**8*). In Fiji alone, ≈500,000 persons were infected, and nearly 50,000 of them became ill (*4**,**7*). The evidence suggests that *Aedes polynesiensis* was the primary vector and that human-mosquito-human transmission predominated without substantial involvement of nonhuman vertebrates in virus amplification (*7*). Once the epidemic ended, Ross River virus evidently disappeared from the region, possibly because of the lack of suitable marsupial reservoir hosts (*7**,**8*). In 1999, a suspected case of Ross River virus disease was reported in a German traveler returning from Fiji and Rarotonga in the Cook Islands (*9*). We present evidence for 2 cases of Ross River virus infection acquired in Fiji in late 2003 and early 2004.

## Case 1

A 39-year-old Canadian man flew to Fiji on October 28, 2003, and returned to Canada on November 10, 2003. Immediately on arrival in Canada, he started experiencing generalized body aches, which lasted until November 17. On November 15, he noticed an erythematous maculopapular rash over his whole body, as well as inguinal lymphadenopathy. The rash and swollen nodes subsided on November 18 and were replaced with the sudden onset of pain and swelling in his right ankle joint and pain without swelling in his right knee and right elbow. Two days later, barely able to walk, the patient sought medical attention at the McGill Centre for Tropical Diseases. He denied any fever or chills and had no history of joint disease. An examination found substantial periarticular tenderness, warmth, erythema, and swelling of his right ankle with essentially full range of motion (Figure). His travel history included an uneventful 4-day trip to Melbourne, Australia, in 1999, involving a trip to the countryside, followed by a week in Bali. In view of his clinical symptoms and recent travel history, a preliminary diagnosis of Ross River virus disease was considered. Laboratory investigations included a complete blood count (CBC); urinalysis; and measurement of levels of liver enzymes, serum creatinine, uric acid, rheumatoid factor, antistreptolysin O, and anti-DNase B, all of which were normal. The erythrocyte sedimentation rate (ESR) was 23 mm/h, and the antinuclear antibody (ANA) test was positive with a speckled pattern. Serum specimens were collected from the patient on days 10, 21, and 141 after the onset of illness; they were screened for elevated immunoglobulin (Ig) M antibody against geographically relevant arboviral antigens by enzyme immunoassays (EIA), when EIA was available for a particular arbovirus. Positive Ross River virus IgM results were then confirmed by plaque reduction neutralization tests. All serologic tests were performed at the arboviral diseases laboratory of the Centers for Disease Control and Prevention (CDC) in Fort Collins, Colorado, as previously described (*10**,**11*). Results of tests for alphavirus antibodies are shown in the Table. Serologic evidence of a dengue infection was absent in both this patient and the patient described in the next section.

**Figure:**
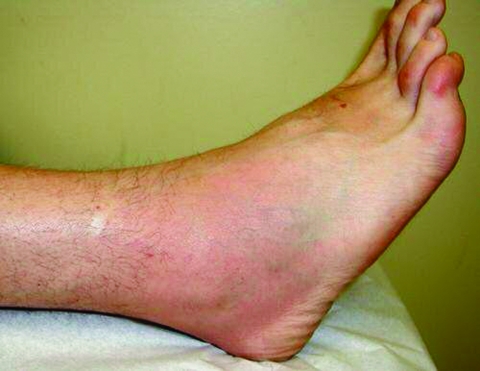
Patient 1: ankle swelling, pain, tenderness, erythema, and warmth on day 10 of illness.

## Case 2

On March 5, 2004, a 44-year-old Canadian woman returned to Canada after visiting New Zealand for 2 months and Fiji for 1 month; she did not travel through Australia or another known Ross River virus–endemic area. She had previously visited Fiji uneventfully in 1995. On March 14, she experienced the abrupt onset of fatigue; the next day she was feverish, nauseated, and anorexic and had severe arthralgia in her ankles and feet. For several days, she experienced extreme hypersensitivity to touch, particularly of her soles, severe enough to prevent weight bearing, and had mild ankle swelling. Her joint pains worsened over several days and spread to her knees, hips, and upper extremities. On March 16, she noticed a nonpruritic, erythematous, maculopapular rash, with small vesicular lesions on the palms, which involved the extremities and face but not the trunk; the rash resolved after 4 days. On March 17, she had normal CBC results and serum creatinine kinase level, mildly elevated liver enzymes, and an ESR of 62 mm/h. By day 10 of illness, she was able to resume limited sedentary work. One month after illness onset, fatigue and joint pain persisted, but physical examination results were normal, apart from difficulties in ambulation due to pain; tests for ANA and rheumatoid factor were negative, C-reactive protein level was normal, and ESR was 30 mm/h. Four months after illness onset, she continued to have gradually resolving arthralgia and fatigue that limited daily activities. At CDC, serologic tests were performed on serum specimens obtained on days 16 and 33 of illness (Table).

**Table:** Results of tests of patients' serum for antibodies to selected alphaviruses*

Patient	Interval (d)§	IgM results†		PRNT titers‡		
		RRV	BFV	RRV	BFV	SINV
1	10	Positive	Negative	320	<10	<10
	21	Positive	Negative	1,280	<10	<10
	141	Equivocal	Negative	160	ND	ND
2	16	Positive	Negative	5,120	<10	ND
	33	Positive	Negative	5,120	<10	ND

*Ig, immunoglobulin; PRNT, plaque-reduction neutralization test; RRV, Ross River virus; BFV, Barmah Forest virus; SINV, Sindbis virus; ND, not done. †IgM-capture enzyme immunoassay; samples tested at 1:400 dilution; positive samples had a positive-to-negative (P/N) absorbance ratio >3.0; equivocal samples had a P/N ratio 2.0–3.0 (*10*); no test for anti-SINV IgM was available. ‡90% plaque-reduction endpoints; >10 is considered positive (*11*). §From onset of illness to serum collection.

## Conclusions

The clinical features and serologic results in these 2 cases provide strong circumstantial evidence for Ross River virus transmission in Fiji during late 2003 and early 2004, which suggests that heightened surveillance is needed as well as epidemiologic and ecologic studies in that region. While both cases were highly clinically compatible with epidemic polyarthritis, and tests for Ross River virus–specific serum IgM antibody were positive in both, the first case is the most convincing serologically because seroconversion (i.e., a 4-fold titer change) in neutralization tests was also observed. The subsequent decrease in this patient's Ross River virus–specific IgM reactivity and neutralizing antibody titer within a few months also argues for a recent Ross River virus infection. In the second case, the high but stable anti-Ross River virus neutralizing antibody titer may reflect the fact that the earliest sample available for testing was obtained >2 weeks after illness onset when the patient's anti–Ross River virus neutralizing antibody titer may have already peaked.

If Ross River virus was circulating in Fiji in 2003 and 2004, at least 2 basic hypotheses may explain its reemergence there. The first of these, which seems the most plausible, involves occasional reintroduction of this virus from the known disease-endemic region (e.g., by viremic persons arriving from Australia), sometimes resulting in local transmission, ultimately followed by local extinction. Circumstantial evidence to support this hypothesis includes the fact that, during the same period that the 2 patients described here traveled to Fiji, Australia was experiencing its usual summer surge in Ross River virus incidence (*3*). The second hypothesis, considered unlikely (*7*), is that Ross River virus became established in Fiji after the 1979–1980 epidemic but remained undetected while causing sporadic and largely unrecognized human cases. No recent serosurveys or other data are available to address this question.

The ability of arboviruses to be moved from one region to another, even from one continent or hemisphere to another, has long been appreciated (*12*). This occurrence may be more frequent than is apparent. Fortunately, the conditions for local transmission and long-term survival of an arbovirus in a new area are often highly complex, so that most such introductions are probably abortive. The recent introduction of West Nile virus to North America and its permanent establishment there, however, is a sobering demonstration that newly introduced arboviruses sometimes achieve long-term survival in new areas where preadapted vectors and suitable vertebrate amplifying hosts are available (*13*). Ross River virus is almost certainly imported into North America fairly frequently because this virus is endemic and often epidemic in Australia, human travel between Australia and North America is frequent, high levels of viremia lasting several days often develop in Ross River virus–infected persons, and cases of Ross River virus disease among visitors to Australia are commonly reported (*14**,**15*). Notably, ≈100 viremic travelers enter New Zealand every year from Queensland alone (*16*). Fortunately, however, to date all such importations into North America evidently have been abortive, and if an introduction of Ross River virus to North America should ever result in local amplification and transmission by preadapted vectors (e.g., *Ae. aegypti* or *Ae. albopictus* [8], activity would probably be short-lived and remain localized, and a lack of optimal vertebrate reservoirs would probably keep the virus from becoming established.

The recent North American experience with West Nile virus, however, emphasizes how uncertain such predictions can be. Therefore, travel medicine specialists and other healthcare providers in North America (and other disease-nonendemic areas) should be familiar with the clinical features of Ross River virus disease, as well as its potential public health importance, and realize that diagnostic tests for this infection currently are available at only a few public health reference laboratories (e.g., CDC).
